# Somatic CRISPR/Cas9-mediated tumour suppressor disruption enables versatile brain tumour modelling

**DOI:** 10.1038/ncomms8391

**Published:** 2015-06-11

**Authors:** Marc Zuckermann, Volker Hovestadt, Christiane B. Knobbe-Thomsen, Marc Zapatka, Paul A. Northcott, Kathrin Schramm, Jelena Belic, David T. W. Jones, Barbara Tschida, Branden Moriarity, David Largaespada, Martine F. Roussel, Andrey Korshunov, Guido Reifenberger, Stefan M. Pfister, Peter Lichter, Daisuke Kawauchi, Jan Gronych

**Affiliations:** 1 Division of Molecular Genetics, German Cancer Research Center (DKFZ), Im Neuenheimer Feld 280, 69120 Heidelberg, Germany; 2 Department of Neuropathology, Heinrich Heine University Düsseldorf, Moorenstrasse 5, 40225 Düsseldorf, Germany; 3 German Cancer Consortium (DKTK), partner site Essen/Düsseldorf, 40225 Düsseldorf, Germany; 4 Division of Pediatric Neurooncology, German Cancer Research Center (DKFZ), Im Neuenheimer Feld 280, 69120 Heidelberg, Germany; 5 Masonic Cancer Center, University of Minnesota, Minneapolis, Minnesota 55455, USA; 6 Tumor Cell Biology, St Jude Children’s Research Hospital, 262 Danny Thomas Place, Memphis, Tennessee 38105, USA; 7 Department of Neuropathology, University of Heidelberg, Im Neuenheimer Feld 220, 69120 Heidelberg, Germany; 8 Clinical Cooperation Unit Neuropathology, German Cancer Research Center (DKFZ), Im Neuenheimer Feld 220-221, 69120 Heidelberg, Germany

## Abstract

*In vivo* functional investigation of oncogenes using somatic gene transfer has been successfully exploited to validate their role in tumorigenesis. For tumour suppressor genes this has proven more challenging due to technical aspects. To provide a flexible and effective method for investigating somatic loss-of-function alterations and their influence on tumorigenesis, we have established CRISPR/Cas9-mediated somatic gene disruption, allowing for *in vivo* targeting of TSGs. Here we demonstrate the utility of this approach by deleting single (*Ptch1*) or multiple genes (*Trp53, Pten, Nf1*) in the mouse brain, resulting in the development of medulloblastoma and glioblastoma, respectively. Using whole-genome sequencing (WGS) we characterized the medulloblastoma-driving *Ptch1* deletions in detail and show that no off-targets were detected in these tumours. This method provides a fast and convenient system for validating the emerging wealth of novel candidate tumour suppressor genes and the generation of faithful animal models of human cancer.

## Abstract (web summary)

Gene transfer is a powerful technique to investigate the mechanistic basis of tumorigenesis. Here Zuckermann *et al*. adapt CRISPR/Cas9 genome editing to target potential oncogenes somatically *in vivo*, establishing a fast and convenient system for validating novel genetic candidates.

## Graphical abstract

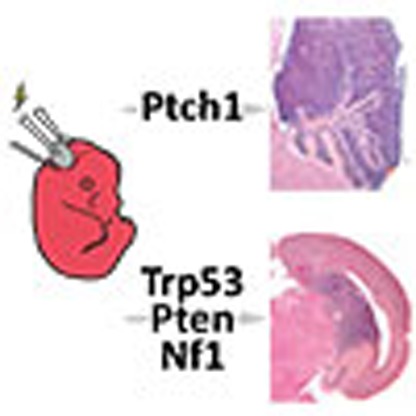

Modelling cancer in mice through engineering of candidate genes in the germline has long been the gold standard for the validation of putative oncogenes or tumour suppressor genes (TSGs). For TSGs, whereby loss-of-function (LOF) mutations act as a driver for malignant transformation, this has traditionally been accomplished using constitutive or cell-type-specific knockout strategies mediated by homologous recombination in embryonic stem cells. Although this allows evaluation of cell-type-specific susceptibility to malignant transformation, generation of genetically engineered mouse models (GEMMs) is a time-consuming process. For *in vivo* investigation of a large number of molecular alterations, such as the many new candidates currently emerging from large-scale tumour genome-sequencing efforts^1^, a faster and more flexible method is required. We therefore sought to adapt the CRISPR/Cas9-guided endonuclease technique^2^,^3^,^4^ for the somatic disruption of candidate TSGs and thereby complement aforementioned already existing models like GEMM. The locus-specific cutting by the Cas9 nuclease is mediated by a guide-RNA (gRNA) encoded on the same vector, targeting a 20mer cognate sequence in the genome. Upon introduction of a double-strand break, erroneous non-homologous end-joining (NHEJ) repair evokes insertions/deletions (indels) and potential frameshift mutations, typically resulting in LOF of the target gene^5^.

While there have been reports of cancer modelling using somatic transfer of CRISPR/Cas9 to the lung by viral-mediated transduction^6^ or to the liver by hydrodynamic injection^7^, here we aimed at using somatic gene transfer of CRISPR/Cas9 to induce brain tumours. We therefore applied polyethylenimine (PEI)-mediated transfection^8^ or *in utero* electroporation of the developing brain^9^. These techniques do not suffer from size limitations applying to viral vectors, are mouse strain independent, provide tissue specificity and yet can be adapted to target a variety of different mouse tissues. We further sought to use these systems to induce specific types of brain tumours.

As a model system, we first focused on sonic hedgehog (SHH) medulloblastoma (MB)^10^, a malignant childhood brain tumour^11^. Genomic studies have delineated four distinct MB subgroups, each with characteristic molecular and clinical features^10^,^12^. SHH subgroup MBs originate from granule neuron precursors (GNPs), a cell type that is exclusively present in the developing cerebellum, and whose proliferation is regulated by the SHH pathway^13^. Germline mouse models that harbour heterozygous deletion of the *Ptch1* gene (i.e., *Ptch1*^*+/−*^), encoding an essential negative regulator of the SHH pathway, develop aberrant clusters of GNPs, so-called ‘preneoplastic hyperplasia’. In 14–20% of these animals, these clusters continue malignant transformation and form full-blown SHH MB within 3 months^14^,^15^. The efficiency of tumor development can be increased by inducing conditional homozygous *Ptch1* deletion in relevant stem/progenitor cell populations^16^,^17^ or by breeding *Ptch1*^*+/−*^ mice to a *Trp53*-null background^18^, which is also seen in a proportion of patients with highly aggressive human SHH MB^19^. Based on this data, we aimed to somatically disrupt *Ptch1* using *in vivo* transfection (P0) and *in utero* electroporation (E13.5) of plasmids encoding both the Cas9 nuclease and a gRNA targeting the endogenous *Ptch1* locus. Using these approaches we were able to induce tumours resembling MB with a high penetrance. Next generation sequencing of these tumours showed large on-site deletions disrupting the *Ptch1* locus whereas no off-target could be detected.

Furthermore, we sought to extend the utility of this approach to glioblastoma (GBM), the most aggressive primary brain tumor^20^. These astrocytic tumours originate from neural stem or glial progenitor cells primarily in the forebrain^21^,^22^ and frequently harbour mutations in the key TSGs *P53, PTEN* and *NF1* (refs 23, 24). Previously, it was shown that *hGFAP-Cre;Nf1*^*f/+*^*;Trp53*^*f/f*^*;Pten*^*f/+*^ mice develop tumours resembling human glioblastoma with high penetrance^25^,^26^. To model these tumours somatically with the CRISPR/Cas9 system, we used *in utero* electroporation of the developing prosencephalon^9^. By delivering three plasmids simultaneously, encoding Cas9 together with gRNAs targeting *Nf1*, *Trp53* and *Pten*, we were able to induce highly aggressive tumours, resembling human GBMs in all of the animals (Fig. 1a).

Collectively, in this study we show that the CRISPR/Cas system can be used to somatically induce LOF mutations in the murine brain leading to the induction of various, specific types of brain tumours with high penetrance. This approach will foster the *in vivo* validation of TSGs identified in recent next generation sequencing studies.

## Results

### Postnatal somatic CRISPR/Cas-mediated deletion of *Ptch1*

**Figure 1:**
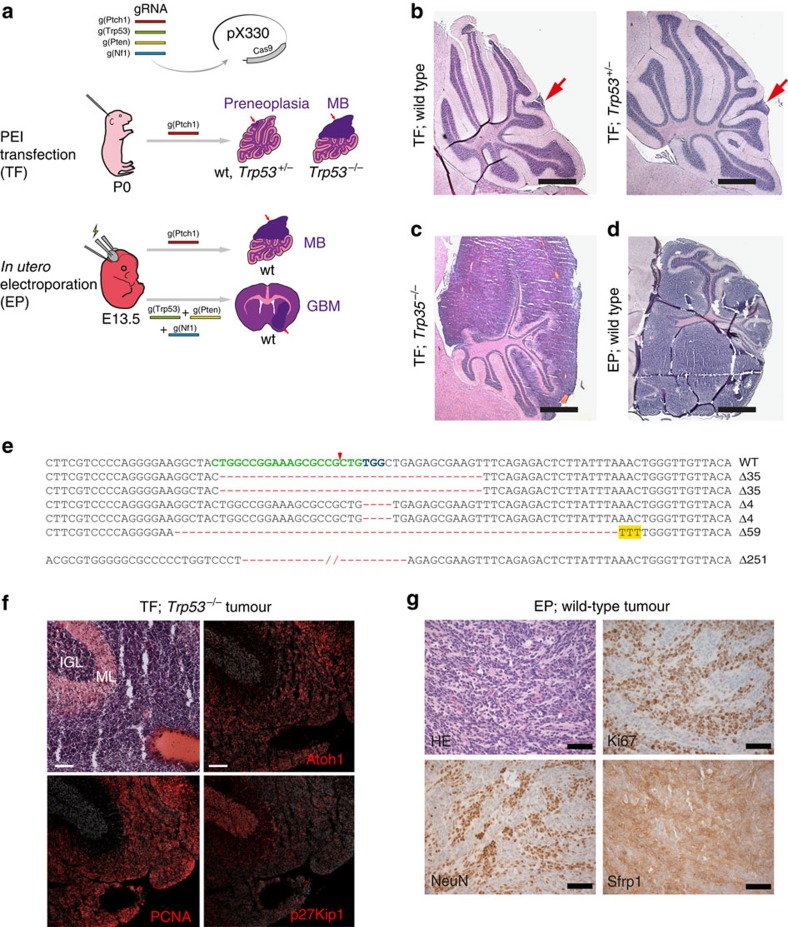
CRISPR/Cas9-mediated somatic deletion of the *Ptch1* locus induces medulloblastoma. (**a**) Cerebella of P0 WT, *Trp53*^+/−^ and *Trp53*^−/−^ mice were transfected with pX330 plasmids encoding Cas9 and a gRNA directed against the *Ptch1* locus. In addition, plasmids were electroporated into cerebellar neuroepithelial cells of WT animals at E13.5. Furthermore, *Trp53*, *Nf1* and *Pten* were targeted simultaneously by electroporation of the ventricular zone of the cerebrum to induce GBM formation. (**b**) Small neoplastic lesions found in WT (*n*=1/14) and *Trp53*^*−/+*^ (*n*=1/11) *gPtch1*/Cas9-transfected animals. The small lesions (arrows) only encompass one cerebellar folium. Scale bars, 200 μm. (**c**) H&E staining of a cerebellar tumour induced by transfection of a *Trp53*^*−/−*^ animal with *gPtch1.1*/Cas9. Scale bar, 200 μm. (**d**) Sagittal section of a WT mouse cerebellum 6 weeks after *in utero* EP of *gPtch1.1*/Cas9 showing a large tumour. Scale bars, 200 μm. (**e**) The targeted locus was amplified from tumour tissue. The PCR products were ligated into a cloning vector and sequenced via Sanger sequencing (*n*=6 clones). Deletions were found in all (6/6) sequences and are displayed as red dashes. Nucleotide exchanges are highlighted in yellow. The gRNA target region is displayed in green, the 3′-PAM sequence in blue. The Cas9 cleavage site is indicated by a red arrowhead. (**f**) H&E and IF staining of cryostat sections of fully developed tumours indicate stronger proliferation (PCNA) and fewer postmitotic cells (p27^Kip1^). Tumours stained positive for the granule cell marker Atoh1. Scale bars, 100 μm. (**g**) Histological (H&E) features and immunohistochemical detection of selected marker proteins (NeuN, Ki67/Mib1 and Sfrp1) of a representative medulloblastoma generated by somatic *Ptch1* disruption. Tumours show densely packed small tumour cells with round to oval, hyperchromatic nuclei and scant cytoplasm, occasionally cells formed neuroblastic rosette-like structures. Mitotic activity was prominent but areas of necrosis were absent. The tumours invaded into the adjacent cerebellar cortex and grew within the meninges along the cerebellar surface (HE). Tumours are immunopositive for NeuN in clusters of more differentiated and less proliferative cells (NeuN). These NeuN-positive cell clusters were intermingled with clusters of NeuN-negative but strongly Mib1-positive proliferative tumour cells (Ki67). Staining for Sfrp1 is homogeneously positive. Scale bars, 50 μm.

We first tested a set of vectors (pX330) carrying *Cas9* and gRNAs targeting *Ptch1 in vitro* using NIH3T3 cells. The two most effective gRNAs as assessed by SURVEYOR assays (designated *Ptch1.1* and *Ptch1.3*, targeting the second exon of *Ptch1*; Supplementary Fig. 1) were used for further *in vivo* analysis. For postnatal delivery of pX330 plasmids targeting *Ptch1* to GNPs, we applied PEI-mediated *in vivo* transfection by stereotactic injection into the neonatal cerebellum (P0) of wild-type (WT) mice. Successful targeting of the cerebellum was confirmed by trypan blue injection (Supplementary Fig. 2a). Expression of transfected plasmids was assessed by co-transfection of a luciferase-encoding plasmid and subsequent bioluminescence imaging (Supplementary Fig. 2b). We confirmed successful indel formation centred around the *gPtch1.1* site *in vivo* at postnatal day P7 by targeted deep sequencing (Supplementary Fig. 3). Despite successful disruption of the *Ptch1* TSG, none of these animals developed MB within a period of 6 months (Table 1). Histologic analysis of brains 8 weeks after birth, however, revealed a small neoplastic lesion characterized by aberrant accumulation of GNP-like cells on the surface of the cerebellum that was restricted to a single folium (Fig. 1b; Table 1). This is consistent with previously published data in which *Ptch1* germline deletion leads to the induction of ‘preneoplastic lesions’ visible 3–6 weeks after birth^27^. These cells were still proliferating, as indicated by immunopositivity for the proliferation marker PCNA (Supplementary Fig. 4).

Germline deletion of the *Trp53* tumour suppressor locus increases the efficiency of SHH MB induction in mice^18^. We therefore PEI-transfected *gPtch1.1*/*Cas9* or *gPtch1.3*/*Cas9* into the developing cerebellum (P0) of *Trp53*^*+/−*^ and *Trp53*^*−/−*^ animals. One of the *Trp53*^*+/−*^ mice showed a small neoplastic lesion similar to the lesion we found in the WT animal (*n*=1/5). The *Trp53*^*−/−*^ animals, however, started to develop neurological symptoms after ∼5 weeks (Supplementary Fig. 5). Post mortem histological assessment revealed presence of tumours resembling the histology of human MB (Fig. 1c). In total we observed tumours in 8/10 animals (Table 1). As controls, we used the pX330 plasmid encoding a non-target gRNA (gNT) together with *Cas9*. None of these animals developed any pre-neoplasia or tumours (Table 1). This demonstrates that postnatal somatic gene transfer using *in vivo* transfection to transiently express Cas9 and a *Ptch1* targeting gRNA is sufficient to induce MB in *Trp53*-knockout mice (‘CRISPR-Ptch1 (TF)’).

### MB induction in WT animals using *in utero* electroporation

Homozygous deletion of *Ptch1* in ventricular zone stem cells (*hGFAP-Cre;Ptch1*^*fl/fl*^) at prenatal stages induces MB with a high penetrance and short latency^16^,^17^. As *gPtch1*/*Cas9* can potentially induce bi-allelic disruption of the *Ptch1* gene in target cells, we sought to enhance MB genesis in WT animals by using *in utero* electroporation with our targeting vector at embryonic day 13.5 (E13.5), when GNPs are born in the upper rhombic lip. To identify the cell types targeted, we used immunofluorescence staining of embryonic brains 2 days after electroporation with a *GFP*-encoding plasmid. Immunofluorescence revealed that Pax2^+^ cells (cerebellar inhibitory interneuron progenitors) and Sox2^+^ cells (neural stem cells) in the ventricular zone as well as Pax6^+^ GNPs in the upper rhombic lip were targeted by *in vivo* electroporation (Supplementary Fig. 6). We selected animals with successful gene transfer by co-electroporating a plasmid encoding a transposon-flanked luciferase gene together with a transposase gene, thereby allowing for intravital bioluminescence imaging (Supplementary Fig. 7). Of animals that were electroporated with *gPtch1*/Cas9 that showed luciferase signal after birth, 11/12 developed tumours within 16 weeks of age (Supplementary Fig. 5, Table 1). After dissection of the brain, a clear enlargement of the cerebellum could be observed (Supplementary Fig. 8). Post mortem analysis using H&E staining revealed the presence of MB encompassing the majority of the cerebellum (‘CRISPR-Ptch1 (EP)’) (Fig. 1d). As controls, we electroporated mice with a gRNA targeting *Trp53* (gTrp53) together with Cas9 but none of these mice developed a tumour (*n*=0/6; Table 1).

To verify intended deleterious *Ptch1* variants induced by *gPtch1*/Cas9, we isolated genomic DNA from the neoplastic lesions and amplified the *Ptch1* locus around the gRNA target site by PCR. Agarose gel electrophoresis showed multiple products of varying size, as compared with the normal control that yielded the expected single band (Supplementary Fig. 9). Sanger sequencing of the subcloned PCR products revealed the presence of deletions ranging from 4 to 251 bp in size. Of note, all of these deletions resulted in a frameshift of the *Ptch1* open-reading frame (Fig. 1e).

We characterized the CRISPR-Ptch1 tumours histologically in more detail to verify their phenotypic similarity to human MB. H&E staining revealed that the small preneoplastic lesions identified in the cerebellum of *gPtch1*/Cas9-injected WT and *Trp53*^*+/−*^ animals were cell-dense, circumscribed lesions and positive for the granule cell marker Pax6^28^ and the postmitotic marker p27^Kip1^ (Supplementary Fig. 10). CRISPR-Ptch1 tumours (TF and EP) were also highly proliferative (PCNA-positive) and positive for the granule cell marker Atoh1^29^, with considerably fewer postmitotic cells (p27^Kip1^) (Fig. 1f). This suggests that both types of lesions were derived from the granule cell lineage but that the tumours had an enhanced proliferative phenotype. Furthermore, the CRISPR-Ptch1 tumours showed a histological appearance reminiscent of nodular (desmoplastic) MB, the prototypic histopathological variant of SHH MB (Fig. 1g). The NeuN-positive cell clusters were intermingled with clusters of NeuN-negative but strongly with Mib1-positive proliferative tumour cells, while staining for Sfrp1, a Shh pathway target and signature gene for the human SHH MB subgroup^30^, was strongly positive. These data show that the generated CRISPR-Ptch1 tumours histologically resemble human SHH MB.

### Molecular characteristics of CRISPR/Cas-induced MB

**Figure 2:**
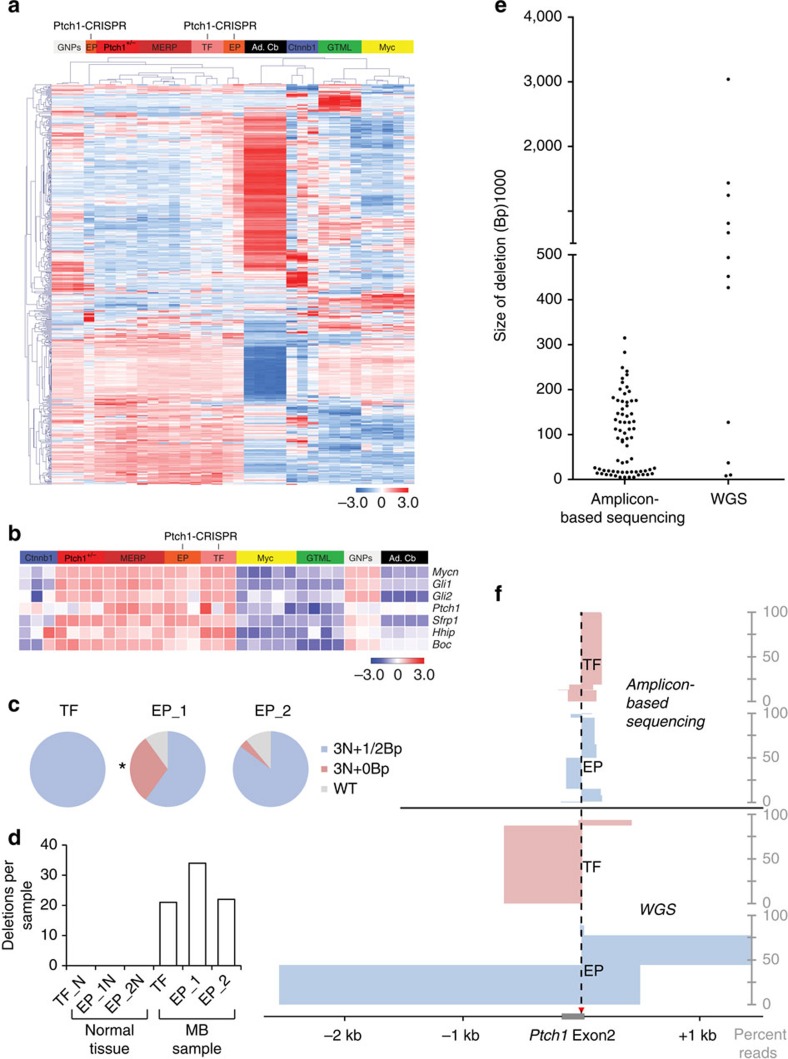
Molecular characterization of CRISPR/Cas9-induced tumours. (**a**) Unsupervised clustering of gene expression data generated from CRISPR-Ptch1 tumours or taken from published data sets of other medulloblastoma mouse models. (**b**) Shh pathway target genes are upregulated in CRISPR-Ptch1 tumours. (**c**) Fraction of frameshift deletions in three different tumour samples. Reads covering the targeted *Ptch1* locus in the WGS data set were analysed for either showing the WT allele, containing a frameshift deletion (deletion size within the exon equals 3 N+1/2 bp) or a deletion that does not result in a frameshift (deletion size equals 3 N+0 bp). The fraction indicated by an asterisk represents a single, big deletion supported by three reads, encompassing 1,435 bp. (**d**) Amount of different *bona fide* deletions found in analysed samples. The targeted *Ptch1* locus of indicated CRISPR-Ptch1 tumours and matched normal tissue was amplified with five different primer pairs and analysed by deep sequencing. A deletion was counted if it ranged >1 bp and was represented in >0.5% of reads in at least two different PCR products. (**e**) Size of deletions found in the *Ptch1* locus in the targeted deep sequencing compared with the WGS data set of the three analysed PTCH1-CRISPR tumours. For the targeted deep sequencing data, a deletion was counted if it ranged >4 bp and was represented in >0.5% of reads in at least two different PCR products. (**f**) Genomic landscape of *Ptch1* deletions found in a representative CRISPR-Ptch1 (TF) (red boxes) and a CRISPR-Ptch1 (EP) (blue boxes) tumour. The targeted region within the *Ptch1* locus is indicated by a red arrow head. The width of each box indicates the size of the deletion, whereas the height indicates the fraction of reads supporting this deletion. For the targeted amplicon-based deep sequencing all deletions were included that occurred in >0.5% of the reads of the PCR product generated with the primers Seq_PTCH_1.1 and Seq_PTCH_1.2. (TF=tumour induced by PEI-mediated transfection with *gPtch1.1*/Cas9, EP=tumour induced by *in utero* electroporation with *gPtch1.1*/Cas9, Myc=Myc T58A plus dominant-negative Trp53 or Myc WT in *Trp53*-null^32^,^33^, GTML=Glt1-TRE;MycN; Luciferase^34^, Ctnnb=*Blbp-Cre*^*+/–*^;*Ctnnb1*^+/lox(ex3)^;*Tp53*^*fl*/fl^^31^, Ad. Cb=adult cerebellum, MERP=*Atoh1-CreERT2;Ptch1*^*fl/fl*^, GNPs=granule neuron precursors).

To further examine the molecular characteristics of somatically induced CRISPR-Ptch1 MBs, we isolated total RNA from six tumours (three *in utero* electroporation on a WT background, three postnatal transfection Trp53^−/−^) and performed array-based gene expression analysis. CRISPR-Ptch1 expression profiles were compared with the respective published profiles of other murine MB models as well as normal adult mouse cerebellum and postnatal murine GNPs. Pairwise unsupervised clustering using a subset of the most variant genes (top 5%, *n*=1,076) showed that both types of CRISPR-Ptch1 tumours are most similar to germline *Ptch1*^*+/−*^ and *Atoh1-CreER*;*Ptch1*^*fl/fl*^^16^ MB models. In contrast, both CRISPR-Ptch1 models are clearly distinct from normal postnatal GNPs or MB mouse models representing Wnt (*Ctnnb1*;*Trp53*)^31^, Group 3 (*Myc*;*Trp53*)^32^,^33^ or Group4 (*Mycn*)^34^ (Fig. 2a). In addition, in both CRISPR-Ptch1 models, a series of SHH target genes (*Mycn*, *Gli1/2*, *Sfrp1*, *Hhip*, *Boc*) showed an increased expression that was comparable to that observed in the other *Ptch1* models and in GNPs (Fig. 2b). Of note, *Ptch1*, which is a Shh target gene itself, can still be detected at an appreciable expression level. However, the observed frameshift mutations are predicted to prevent expression of a functional protein as indicated by Shh pathway activation in these tumours. These data clearly demonstrate that our somatically induced MBs histologically and molecularly resemble MB of the relevant subgroup.

To analyse the indels generated by somatic gene transfer of *gPtch1*/*Cas9* in greater detail, we performed targeted ultra-deep sequencing of the *Ptch1* locus in three different tumours (two CRISPR-Ptch1 (EP), one CRISPR-Ptch1 (TF); Supplementary Table 1) and matched normal tissue using five different staggered amplicons (251 bp paired-end, on average >200,000-fold coverage per amplicon; Supplementary Data 1). In addition, to capture the full picture of genomic variants around the *Ptch1* locus, we performed WGS of the same six samples (101 bp paired-end, on average 23.3-fold coverage; Supplementary Data 2). In all three MBs, non-frameshift indels as well as the WT *Ptch1* locus were highly underrepresented or absent in the WGS data (Fig. 2c). This indicates that both *Ptch1* alleles were disrupted in the tumours. In the targeted sequencing data, we identified 21, 22 and 34 different indels per tumour sample (>1 bp; represented in >0.5% of reads in at least two different amplicons), indicating a high degree of polyclonality. The majority of sequencing reads (>75%) were representing at most three indels per sample, though, which indicates the existence of few predominant clones (Fig. 2d). Interestingly, even indels with the highest read-score in this data set were underrepresented in the WGS data, indicating that most of the deletions induced by *gPtch1*/Cas9 could not be detected with amplicon-based sequencing approaches (Fig. 2e,f).

Our WGS data also facilitated a genome-wide analysis of potential off-target effects of *gPtch1*/*Cas9* in the aforementioned tumours. To firstly perceive a full picture of genomic variants, we applied a permissive filter setting and obtained 122,364 deletions in all of the six analysed samples in total. However, none of these mapped with the sequence of our *Ptch1* gRNA even when allowing for up to three mismatches, besides the deletions in *Ptch1* itself (Supplementary Data 3). When filtering for loci that were recurrently mutated in all three samples we found only *Ptch1* as *bona fide* hit (Supplementary Data 4). To look for potential off-target effects in more detail, we filtered for loci displaying different deletions in one sample and again only found *Ptch1* as plausible hit (Supplementary Data 5). Thus, these data indicate no detectable off-targets in this setting.

### GBM induced by multiplex gene targeting

**Figure 3:**
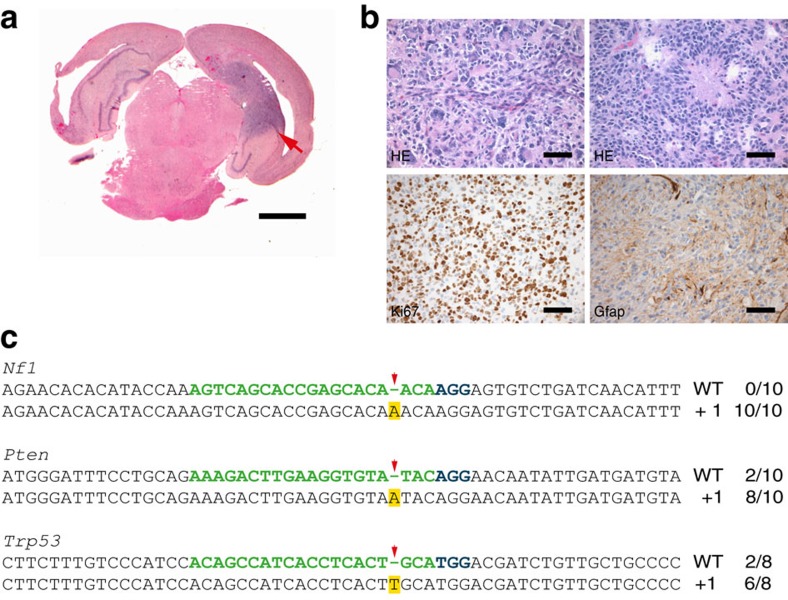
Simultaneous targeting of Nf1, Pten and Trp53 loci in the forebrain induces GBM. (**a**) H&E-stained coronal section of the brain of a WT mouse 8 weeks after *in utero* EP of *gTrp53*, *gPten*, *gNf1*/Cas9 showing a tumour in one hemisphere (arrow). Scale bar, 2 mm (**b**) H&E (left panel): cellular tumour with pleomorphic often multinucleated tumour cells and pathological vascular proliferations. H&E (right panel): staining of the same cellular tumour displaying focal necroses with pseudopalisading of tumour cells. Tumour cells are highly proliferative as indicated by Ki67 stianing. GFAP staining showing a moderate to strong labelling of the majority of the tumour cells. Scale bars, 50 μm. (**c**) The targeted loci were PCR-amplified from tumour tissue. The PCR products were ligated into a cloning vector and sequenced via Sanger sequencing (number of clones indicated). Nucleotide insertions are highlighted in yellow. The gRNA target regions are displayed in green, the 3′-PAM sequences in blue. The Cas9 cleavage sites are indicated by a red arrowhead.

To extend the utility of this approach to other tumour entities and multiple gene targets, we sought to induce glioblastoma (GBM), the most aggressive primary brain tumour^20^, by simultaneously disrupting multiple TSGs using *in utero* electroporation. The high penetrance of MB formation demonstrated the efficiency of this method for delivering expression plasmids into the developing brain. Previously, it was shown that *hGFAP-Cre*;*Nf1*^*f/+*^;*Trp53*^*f/f*^;*Pten*^*f/+*^mice develop GBMs with high penetrance^25^. We therefore electroporated the ventricular zone of the cerebrum in E13.5 WT embryos with plasmids carrying Cas9 and gRNAs directed against endogenous *Trp53*, *Pten* and *Nf1* loci (*gTrp53*, *gPten*, *gNf1*). By 6 weeks of age the first animals began to show neurological symptoms (Supplementary Fig. 5). Post mortem histologic analysis revealed the presence of tumours in the forebrain with histologic features resembling GBM (Fig. 3a,b). In total 8/8 animals exhibited tumours after 6–14 weeks, whereas all animals injected with either gRNA alone did not show any tumour-related symptoms (Table 2). Clonal Sanger sequencing of the three target loci revealed indels resulting in frameshifts in each of the respective genes (Fig. 3c). These data show that intracerebral somatic gene transfer of CRISPR/Cas9 can induce specific tumours via the simultaneous targeting of multiple TSGs.

## Discussion

The validation of the oncogenic role of mutations identified in human tumours and the generation of animal models faithfully recapitulating the molecular, biological and clinical characteristics of their human counterparts are key aspects in translational oncology. Genetically engineered mouse models have contributed significantly to the better understanding of tumour biology and the preclinical testing of novel treatment approaches. However, the elaborate generation of GEMMs cannot keep pace with the ever increasing wealth of data resulting from cancer genome-sequencing studies. Therefore, we set out to combine somatic gene transfer and recent developments in the field of guided nucleases to somatically disrupt TSGs in the murine brain and thereby induce different types of brain tumours. We used postnatal PEI-mediated transfection and *in utero* electroporation and focused on MB, an embryonal tumour mainly occurring in young patients, and GBM, the most malignant primary astrocytic brain tumour.

Targeting *Ptch1* by PEI-mediated postnatal transfection of wild-type mice or mice carrying a homozygous deletion of *Trp53* evoked preneoplastic lesions or full-blown MB, respectively. This is consistent with previously published data, in which a *Trp53* deletion can accelerate tumorigenesis in *Ptch1* heterozygous GEMMs^18^. It had been demonstrated that bi-allelic *Ptch1* deletion at embryonic stages can enhance tumorigenesis in *Ptch1*-driven MB models^16^,^17^. By targeting *Ptch1* via *in utero* electroporation, we did not require additional oncogenic hits for MB induction. Therefore, our somatic gene transfer-based MB models recapitulate many features that had already been observed using genetically engineered mice.

By targeting *Nf1*, *Pten* and *Trp53* simultaneously in the forebrain of E13.5 mice, we were able to induce highly aggressive tumours resembling human GBMs. Thereby, we could show that the somatic CRISPR/Cas-mediated tumour induction via *in utero* electroporation is compatible with multiplexing and modelling of other brain tumour entities. While multiplexing has already been shown using viral gene transfer to the liver and lung^6^,^7^, we here demonstrate that *in utero* electroporation can be used as a non-viral approach to target the brain, even at embryonic stages, which further complements the utility of somatic CRISPR/Cas9-mediated genome editing.

We analysed the tumours induced by somatic *Ptch1* deletion using amplicon-based sequencing and whole-genome sequencing (WGS). In these analyses, the wild-type allele was highly underrepresented indicating a biallelic disruption of the locus. We also investigated further characteristics of the induced indels and could show that many of the deletions we found were larger than 500 bp (*n*=6/13, Fig. 2e,f). Deletions of this size have scarcely been described as a result of CRISPR/Cas9-induced double-strand breaks so far^2^,^3^,^35^,^36^, which might partially owe to the limitations of amplicon-based sequencing methods that have been predominantly used in these studies. Detected insertions were much smaller in size (<10 bp). As we show here, WGS can improve the detectable resolution of variants induced by CRISPR/Cas that might aid downstream data interpretation in similarly focused studies. Compared with the big deletions we found in the *Ptch1* locus of CRISPR-PTCH1 tumours, the small insertions identified in the induced GBMs hint towards a very variable repair of CRISPR-induced double-strand breaks *in vivo*, potentially specific for the respective cell type or genomic locus. However, further studies will be needed to elucidate the underlying repair mechanisms.

The WGS data of the CRISPR-PTCH1 tumours facilitated us to investigate the occurrence of recurrent off-target indels. With the applied coverage and filters, no recurrent off-target deletions could be identified. Although we cannot exclude the well described^37^,^38^,^39^,^40^ Cas9 off-target activity in general, no clonal enrichment of the respective indels could be detected in this setting.

Taken together, we demonstrate that somatic gene transfer of CRISPR plasmids encoding Cas9 and gRNAs directed against single or multiple TSGs is suitable to induce distinct brain tumour entities. Furthermore, these tumours recapitulate the phenotype of the corresponding germline-based animal models and do not carry detectable off-target deletions. Thus, this approach represents a fast and versatile method to investigate larger sets of candidate TSGs *in vivo* not only for brain tumours but also for other cancers.

## Methods

### Vector construction

Oligonucleotides coding for guide RNAs that target the second exon of the *Ptch1* locus were constructed and cloned into pX330 (Addgene) according to the original online protocol of the Zhang lab (http://www.genome-engineering.org/crispr/wp-content/uploads/2014/05/CRISPR-Reagent-Description-Rev20140509.pdf). In this study, the following 20 bp sequences were targeted: Ptch1.1 5′-CTGGCCGGAAAGCGCCGCTG-3′, Ptch1.2 5′-TCAGAGACTCTTATTTAAAC-3′, Ptch1.3 5′-GTTGTGGGTCTCCTCATATT-3′, Nf1 5′-AGTCAGCACCGAGCACAACA-3′, Pten 5′-AAAGACTTGAAGGTGTATAC-3′ and Trp53 5′-ACAGCCATCACCTCACTGCA-3′. For control experiments, a gRNA targeting the following 20 bp sequence was designed: 5′-GCGACCAATACGCGAACGTC-3′. The sequence does not map to the most recent mouse genome assembly (mm10) without base change or when changing any one or two positions, and maps three times when changing any three positions (analysed using SeqMap v1.0.13 (ref. 41)). For luciferase imaging of P0-transfected mice, the vector pT2C-LucPGK-SB100X was used. To control for successful electroporation the plasmids pT2K IRES-luciferase and pCAGGS-T2TP^43^ were co-electroporated. For the construction of pT2K IRES-luciferase the gene encoding IRES-luciferase was inserted into EcoRI and XhoI sites of pT2K CAGGS^43^.

### Surveyor assay

A total of 2 × 10^5^ NIH3T3 cells (obtained from ATCC, authenticated as murine and mycoplasma free) were seeded in six-well dishes containing 2 ml IMDM medium (Life Technologies; supplemented with FCS, penicillin/streptavidin and glutamine) per well 1 day before transfection. Lipofectamine 2,000 (Life Technologies) was used to transfect cells with 3 μg Cas9/gRNA expression vector according to the manufacturer’s instructions. DNA was isolated with the QiaAMP DNA Mini Kit (Qiagen) at 3 days post transfection. The respective locus was amplified using the primers Survey_PTCH_fwd 5′-GGATGGTCCTGGTTCCAGTC-3′ and Survey500_PTCH_rev 5′-CCCGAGTAGATTACAGCGCG-3′ with PRECISOR polymerase (Biocat) in GC buffer (Biocat). Heterodimerization and digestion with SURVEYOR nuclease were performed with the SURVEYOR Mutation Detection Kit (Transgenomic) according to the manufacturer’s instructions. Cleavage products were separated on a 2% agarose gel and stained with ethidium bromide for 10 min. Images were captured with the Gel Doc system (Bio-Rad). Band intensities were quantified via ImageJ. Gene modification levels were calculated using the following equation^44^: % genes modified=(1−(1−fraction cleaved)^0.5^ ) × 100.

### Animal studies

For *in vivo* transfection plasmid vectors were purified using the EndoFree Plasmid Maxi Kit (Qiagen). A mix of plasmid DNA and *in vivo*-jetPEI transfection reagent (Polyplus-transfection) was prepared according to the manufacturer’s instructions. P0 C57BL/6N mice (male and female) were anaesthetised with 2% isoflurane and medially injected at lambda: −3.6 and D/V: −0.7 with 1 μl of either DNA transfection reagent mix or trypan blue using a 75 RN Neuros Syringe and a 33-gauge needle (Hamilton). For *in utero* electroporation, surgical operation and electroporation-based gene transfer was performed as follows. After injection of DNA plasmids (1 μl, 2.5 g l^−1^ in PBS) into the fourth ventricle (4th V.) or cerebral ventricular zone (VZ) of E13.5 Crl:CD1(ICR) embryos, electric square pulses were delivered laterally using forceps-like electrodes (32 mV (4th V.) or 35 mV (VZ), 50ms-on, 950ms-off, 5 pulses). The operated embryos were allowed to survive until being born and were subjected to bioluminescence imaging of luciferase activity. All animal experiments have been performed according to animal welfare regulations and have been approved by the responsible authorities (Regierungspräsidium Karlsruhe, approval numbers G-98/13; G-176/13; G-209/14).

### Luciferase imaging

For mice that were co-transfected with luciferase-encoding vector pT2C-LucPGK-SB100X at P0, imaging was performed 2 days post transfection. Electroporated mice were monitored in the first postnatal week. Luciferase signals were captured using the Xenogen IVIS-100 Imaging System. Mice were anaesthetized with 2% isoflurane and injected with 3 mg (P7) or 200 μg (P2) of StayBrite D-Luciferin (Biocat) intraperitoneally 5–7 min before the imaging. Images were acquired and radiance (photons persec percm^2^ persteradian) was determined within regions of interest (mouse head). Only neonates whose brain carried luciferase signals were selected for further analysis.

### Histological analysis

For histological and immunohistochemical analyses, brains from treated mice were fixed in 4% phosphate-buffered formaldehyde, dehydrated in a STP 120 spin tissue processor (Thermo Scientific) and embedded in paraffin. Sections (5 μm thickness) were cut and mounted onto glass slides, deparaffinized in xylene and rehydrated in a descending series of alcohols, then rinsed in distilled water and stained. Haematoxylin-eosin (HE) staining was carried out according to routine laboratory protocols. For immunohistochemical stainings, enzyme-induced antigen retrieval (for anti-GFAP) using pepsin (1% in 0.01 M HCl) or heat-induced antigen retrieval (all other antibodies) using a 1 × target retrieval solution (Dako) was performed. Primary antibodies used were rabbit anti-GFAP (Dako Z0334, 1:1,000), rabbit anti-Ki67 (CellMarque, Rocklin, CA, 275R-14, 1:200), mouse anti-NeuN (Merck Millipore, Billerica, MA, MAB377, 1:200) and rabbit anti-SFRP1 (Abcam, ab4193, 1:200). Secondary antibodies were diluted 1:500 (anti-mouse, VectorLab, BA-2,000 or anti-rabbit, VectorLab, BA-1,000) and staining was visualized using the avidin-biotin peroxidase system (VectorLab) and freshly prepared diaminobenzidine as chromogen (Dako). Slides were counterstained with haematoxylin, dehydrated and mounted.

### Immunofluorescence studies

Cerebella of mice were formalin-fixed and paraffin-embedded (8 μm sections; see above). Alternatively, brains were fixed with 4% PFA in PBS for 2.5 h on ice, followed by cryoprotection with 30% sucrose in PBS overnight. Fixed tissues were embedded in O.C.T Compound (Sakura), frozen on dry ice and cryopreserved at −80 °C for cryosections (12 μm thickness). For staining with an anti-PCNA antibody, sections were subjected to heat antigen retrieval with 10 mM citrate buffer for 30 min. After blocking with 10% normal donkey serum in PBS-T (0.1% Triton X-100 in PBS) for 30 min at room temperature, sections were incubated with primary antibody overnight at 4 °C. Incubation with secondary antibody was performed for 30 min at room temperature. Subsequently, sections were mounted using the ProLong Gold antifade reagent (Lifetechnologies). The antibodies used for IHC were anti-Pax6 (1:1,000, Covance, PRB-278P), anti-PCNA (1:1,000, Merck Millipore, NA03), anti-GFP (1:1,000, Abcam, ab13970), anti-GFP (1:1,000, Cell Signaling, 2,555), anti-Pax2 (1:1,000, Invitrogen, 71-6000), anti-Calbindin (1:1,000, Millipore, AB1778), anti-Sox2 (1:500, Santa Cruz, sc-17320), anti-p27 (1:1,000, BD Biosciences, 610242) and anti-Atoh1 (1:1,000, Muguruma *et al*.^42^).

### Sanger sequencing

Tumour tissue or normal brain tissue was scraped from H&E-stained sections and the DNA was isolated using the DNeasy Blood & Tissue Kit (Qiagen). The respective loci were amplified using the primers Seq_PTCH_2.1 5′-GCACCCCAAGTCTCATTCAG-3′ and Seq_PTCH_2.2 5′-GTCTCGAGATTAGCTGCCTTT-3′, Seq_Pten_1.1 5′-GCCTCAGTCGCGTATTCTG-3′ and Seq_Pten_1.2 5′-CATCCAGTGACGCATCCAG-3′, Seq_P53_1.1 5′-CAGGGTCTCAGAAGTTTGAGG-3′ and Seq_P53_1.2 5′-GCATTGAAAGGTCACACGAAAG-3′ or Seq_Nf1_1.1 5′-GGAAAACACTGGATAGAAGATTTG-3′ and Seq_Nf1_1.2 5′-GCTTCAACGGGAATAAAAACCTG-3′. The PCR products were separated on a 2% agarose gel, stained with ethidium bromide for 10 min and analysed with the Gel Doc system (Bio-Rad). Subsequently PCR-products of tumor tissue were cloned into pJET1.2 (Thermo Scientific). Plasmids of 6–10 colonies per analysed locus were subjected to sequencing with an ABI PRISM 7900HT Sequence Detection System (Applied Biosystems).

### Targeted deep sequencing

DNA from five frozen whole cerebella and three tumours was isolated using the DNeasy Blood & Tissue Kit (Qiagen). As controls, DNA from corresponding fresh frozen mouse tails was isolated via standard phenol/chloroform extraction. The Ptch1 locus of each sample was amplified with the following five consecutively shifted primer pairs; Seq_PTCH_1.1 5′-CTCACTGATTTACAACCAAGGC-3′ and Seq_PTCH_1.2 5′-CATCAAACACAGTAAAGGGAAGG-3′; Seq_PTCH_2.1 5′-GCACCCCAAGTCTCATTCAG-3′ and Seq_PTCH_2.2 5′-GTCTCGAGATTAGCTGCCTTT-3′; Seq_PTCH_3.1 5′-CACACTGCTGTCCAGAGGG-3′ and Seq_PTCH_3.2 5′-ACTGGCCAGCCATGCAAAC-3′; Seq_PTCH_4.1 5′-CCTGGTCCCTAGAGTACAG-3′ and Seq_PTCH_4.2 5′-CGCCTGATCGCTTACCTTC-3′; Seq_PTCH_5.1 5′-GGTTCCAGTCCGAGGGAG-3′ and Seq_PTCH_5.2 5′-CAAGGGCTTCTCGTTGGCTA-3′. To control for a successful PCR, 1/10 of PCR products were analysed on a 2% agarose gel. For each sample, the remainder of the 5 PCR products was pooled and purified with the QIAquick PCR Purification Kit (Qiagen). After library preparation, barcoded sequencing was performed using the Illumina MiSeq platform, yielding over 1.2 million 251 bp paired-end reads per sample. Subsequently, reads were sorted into amplicons according to the presence of the primer sequences at the beginning of both ends, not allowing for any mismatches. Both ends were merged using Pear v0.9.4 (ref. 45) and the following non-default parameter: *P*value 0.0001. Resulting fragments of the same sequence were combined, and fragments occurring <100 times were removed. If multiple fragments of the same length per sample and amplicon were present, the sequence occurring most frequently was retained as the predominant sequence. For non-reference fragments insertions and deletions were annotated in detail. Indels were called if present in at least two amplicons and making up for at least 0.5% of the reads. Indel length is given as the length of the affected reference sequence minus the length of the alternative sequence.

### Whole-genome sequencing

DNA of the same tumour and control samples were used for WGS. After library preparation, sequencing was performed using the Illumina HiSeq platform, yielding on average 330 million 101 bp paired-end reads per sample. Sequences were aligned to the mm10 reference genome retrieved via the UCSC browser using the ‘mem’ algorithm of bwa version 0.7.10 [Li (2013) arXiv:1303.3997] and default settings. Alignments were coordinate-sorted and merged using Picard. For the downstream analysis of indels, both ends were treated individually and only reads containing at least two mapping stretches (CIGAR code M) separated by stretches of insertions or deletions, as well as split reads mapping to the same chromosome, were retained. Indels were extracted using a custom R-script, making use of the GenomicAlignments BioConductor package. Individual reads containing indels were retained whether each mapping stretch was at least 25 bp in size, and at least 95 bp were mapped in total. Individual reads indicating the same indel were combined, while filtering putative PCR duplicates. On the basis of results from the targeted sequencing, resulting deletions were retained if being between 4 bp and 100 kb in size. In addition, both start and end position of deletions were required to not overlap start or end positions of deletions in the combined control samples (extended by 5 bp), or share >80% identity with known polymorphic indels in mouse^46^. Indels were further filtered if start or end positions (extended by 5 bp) overlapped annotated simple repeats (RepeatMasker, retrieved via the UCSC browser) or regions with a combined coverage larger than 250 in the control samples. All WGS data were re-aligned to a reference set comprising the resulting sequences of the indels identified by targeted sequencing or indels affecting the Ptch1 locus in the genome-wide WGS analysis.

### Gene expression analysis

Published gene expression array profiles for murine Myc-driven^32^ and Wnt-driven^47^ MBs were accessed from the Gene Expression Omnibus under the accession numbers GSE33199 and GSE24628, respectively. Total RNA was extracted from murine cerebellar tumours, non-neoplastic GNPs and normal adult mouse cerebellum, and hybridized to Affymetrix Mouse Genome 430 2.0 arrays at the Genomics and Proteomics Core Facility of the DKFZ according to the manufacturer’s instructions. Affymetrix CEL files were processed using Affymetrix Expression Console (v1.3.1) and normalized signal intensities were generated using the RMA algorithm. Two-dimensional unsupervised hierarchical clustering (Pearson Correlation, average linkage) of tumour and normal gene expression profiles was performed using the top 5% of high s.d. genes (*n*=1,076) across the samples. Clustering results and expression heat maps were generated using the TM4 MultiExperiment Viewer^48^.

## Additional information

**Accession codes**: Sequencing data associated with this study were deposited in the European Nucleotide Archive under accession code PRJEB6541. Gene expression profiles associated with this study were deposited in the Gene Expression Omnibus under accession code GSE58629.

**How to cite this article:** Zuckermann, M. *et al*. Somatic CRISPR/Cas9-mediated tumour suppressor disruption enables versatile brain tumour modelling. *Nat. Commun*. 6:7391 doi: 10.1038/ncomms8391 (2015).

## Supplementary Material

Supplementary Figures and Table Supplementary Figures 1-10 and Supplementary Table 1

Supplementary Data 1 Deep sequencing of the Ptch1 locus - all indels

Supplementary Data 2 Indels found by WGS and by targeted deep sequencing in the Ptch1 locus

Supplementary Data 3 All filtered indels of WGS dataset

Supplementary Data 4 Recurrent mutation in WGS dataset

Supplementary Data 5 Loci displaying different deletions in one sample identified by WGS

## Figures and Tables

**Table 1:** Incidences of cerebellar tumours.

gRNA	Method	Genotype *Trp53*	Tumour incidence	Killed (age in weeks) to check for tumour development
*gPtch1.1*	TF	WT	1/14*	0/14
*gPtch1.1*	TF	+/−	1/8*	2/8(6w), 2/8(8w)
*gPtch1.1*	TF	−/−	5/5	1/5(8w)
*gPtch1.3*	TF	WT	0/0	0/0
*gPtch1.3*	TF	+/−	0/3	0/3
*gPtch1.3*	TF	−/−	3/5	2/5(11w)
*gNT*	TF	WT	0/4	0/4
*gNT*	TF	+/−	0/2	2/2(7w)
*gNT*	TF	−/−	0/3	2/3(7w)
*gPtch1.1*	EP	WT	7/7	1/7(7w)
*gPtch1.3*	EP	WT	4/5	0/5
*gTrp53*	EP	WT	0/6	0/6

Abbreviations: EP, tumour induced by *in utero* electroporation with *gPtch1.1*/Cas9; gNT, negative control gRNA without genomic target; gRNA, guide-RNA; TF, tumour induced by PEI-mediated transfection with *gPtch1.1*/Cas9; w, weeks; WT, wild type. Mice were killed after control intervals (age indicated), when presented with neurological symptoms or at 6 months of age. Mice counted as having no tumour displayed histology without pathological findings and/or did not show neurological symptoms by 6 months of age.

^*^Small neoplastic region only.

**Table 2:** Incidences of forebrain tumours.

gRNA	Tumour incidence
*gTrp53*, *gNf1*, *gPten*	8/8
*gTrp53*	0/3
*gNf1*	0/4
*gPten*	0/6

Abbreviation: gRNA, guide-RNA. All constructs were delivered by *in utero* electroporation of E13.5 WT animals. Mice counted as having no tumour displayed no neurological symptoms by 6 months of age.

## References

[b1] Garraway L. A. & Lander E. S. Lessons from the cancer genome. Cell 153, 17–37 (2013). 23540688 10.1016/j.cell.2013.03.002

[b2] Cong L. . Multiplex genome engineering using CRISPR/Cas systems. Science 339, 819–823 (2013). 23287718 10.1126/science.1231143 PMC3795411

[b3] Mali P. . RNA-guided human genome engineering via Cas9. Science 339, 823–826 (2013). 23287722 10.1126/science.1232033 PMC3712628

[b4] Jinek M. . A programmable dual-RNA-guided DNA endonuclease in adaptive bacterial immunity. Science 337, 816–821 (2012). 22745249 10.1126/science.1225829 PMC6286148

[b5] Valerie K. & Povirk L. F. Regulation and mechanisms of mammalian double-strand break repair. Oncogene 22, 5792–5812 (2003). 12947387 10.1038/sj.onc.1206679

[b6] Platt R. J. . CRISPR-Cas9 knockin mice for genome editing and cancer modeling. Cell 159, 440–455 (2014). 25263330 10.1016/j.cell.2014.09.014 PMC4265475

[b7] Xue W. . CRISPR-mediated direct mutation of cancer genes in the mouse liver. Nature 514, 380–384 (2014). 25119044 10.1038/nature13589 PMC4199937

[b8] Abdallah B. . A powerful nonviral vector for *in vivo* gene transfer into the adult mammalian brain: polyethylenimine. Hum. Gene Ther. 7, 1947–1954 (1996). 8930654 10.1089/hum.1996.7.16-1947

[b9] Saito T. In vivo electroporation in the embryonic mouse central nervous system. Nat. Protoc. 1, 1552–1558 (2006). 17406448 10.1038/nprot.2006.276

[b10] Taylor M. D. . Molecular subgroups of medulloblastoma: the current consensus. Acta. Neuropathol. 123, 465–472 (2012). 22134537 10.1007/s00401-011-0922-z PMC3306779

[b11] Ostrom Q. T. . CBTRUS statistical report: Primary brain and central nervous system tumors diagnosed in the United States in 2006-2010. Neuro-oncol. 15, (Suppl 2): 1–56 (2013). 24137015 10.1093/neuonc/not151 PMC3798196

[b12] Northcott P. A. . Medulloblastomics: the end of the beginning. Nat. Rev. Cancer 12, 818–834 (2012). 23175120 10.1038/nrc3410 PMC3889646

[b13] Wechsler-Reya R. J. & Scott M. P. Control of neuronal precursor proliferation in the cerebellum by Sonic Hedgehog. Neuron 22, 103–114 (1999). 10027293 10.1016/s0896-6273(00)80682-0

[b14] Goodrich L. V., Milenkovic L., Higgins K. M. & Scott M. P. Altered neural cell fates and medulloblastoma in mouse patched mutants. Science 277, 1109–1113 (1997). 9262482 10.1126/science.277.5329.1109

[b15] Wetmore C., Eberhart D. E. & Curran T. The normal patched allele is expressed in medulloblastomas from mice with heterozygous germ-line mutation of patched. Cancer Res. 60, 2239–2246 (2000). 10786690

[b16] Yang Z. J. . Medulloblastoma can be initiated by deletion of Patched in lineage-restricted progenitors or stem cells. Cancer Cell 14, 135–145 (2008). 18691548 10.1016/j.ccr.2008.07.003 PMC2538687

[b17] Schuller U. . Acquisition of granule neuron precursor identity is a critical determinant of progenitor cell competence to form Shh-induced medulloblastoma. Cancer Cell 14, 123–134 (2008). 18691547 10.1016/j.ccr.2008.07.005 PMC2597270

[b18] Wetmore C., Eberhart D. E. & Curran T. Loss of p53 but not ARF accelerates medulloblastoma in mice heterozygous for patched. Cancer Res. 61, 513–516 (2001). 11212243

[b19] Kool M. . Genome sequencing of SHH medulloblastoma predicts genotype-related response to smoothened inhibition. Cancer Cell 25, 393–405 (2014). 24651015 10.1016/j.ccr.2014.02.004 PMC4493053

[b20] Gonzales M. The 2000 World Health Organization classification of tumours of the nervous system. J. Clin. Neurosci. 8, 1–3 (2001). 10.1054/jocn.2000.0829 11148073

[b21] Zong H., Parada L. F. & Baker S. J. Cell of origin for malignant gliomas and its implication in therapeutic development. Cold Spring Harb. Perspect. Biol. 7, a020610 (2015). 25635044 10.1101/cshperspect.a020610 PMC4448618

[b22] Alcantara Llaguno S. . Malignant astrocytomas originate from neural stem/progenitor cells in a somatic tumor suppressor mouse model. Cancer Cell 15, 45–56 (2009). 19111880 10.1016/j.ccr.2008.12.006 PMC2650425

[b23] Verhaak R. G. . Integrated genomic analysis identifies clinically relevant subtypes of glioblastoma characterized by abnormalities in PDGFRA, IDH1, EGFR, and NF1. Cancer Cell 17, 98–110 (2010). 20129251 10.1016/j.ccr.2009.12.020 PMC2818769

[b24] Brennan C. W. . The somatic genomic landscape of glioblastoma. Cell 155, 462–477 (2013). 24120142 10.1016/j.cell.2013.09.034 PMC3910500

[b25] Chen J. . A restricted cell population propagates glioblastoma growth after chemotherapy. Nature 488, 522–526 (2012). 22854781 10.1038/nature11287 PMC3427400

[b26] Zhu Y. . Early inactivation of p53 tumor suppressor gene cooperating with NF1 loss induces malignant astrocytoma. Cancer Cell 8, 119–130 (2005). 16098465 10.1016/j.ccr.2005.07.004 PMC3024718

[b27] Oliver T. G. . Loss of patched and disruption of granule cell development in a pre-neoplastic stage of medulloblastoma. Development 132, 2425–2439 (2005). 15843415 10.1242/dev.01793

[b28] Duan D., Fu Y., Paxinos G. & Watson C. Spatiotemporal expression patterns of Pax6 in the brain of embryonic, newborn, and adult mice. Brain Struct. Funct. 218, 353–372 (2013). 22354470 10.1007/s00429-012-0397-2

[b29] Akazawa C., Ishibashi M., Shimizu C., Nakanishi S. & Kageyama R. A mammalian helix-loop-helix factor structurally related to the product of Drosophila proneural gene atonal is a positive transcriptional regulator expressed in the developing nervous system. J. Biol. Chem. 270, 8730–8738 (1995). 7721778 10.1074/jbc.270.15.8730

[b30] Northcott P. A. . Medulloblastoma comprises four distinct molecular variants. J. Clin. Oncol. 29, 1408–1414 (2011). 20823417 10.1200/JCO.2009.27.4324 PMC4874239

[b31] Gibson P. . Subtypes of medulloblastoma have distinct developmental origins. Nature 468, 1095–1099 (2010). 21150899 10.1038/nature09587 PMC3059767

[b32] Kawauchi D. . A mouse model of the most aggressive subgroup of human medulloblastoma. Cancer Cell 21, 168–180 (2012). 22340591 10.1016/j.ccr.2011.12.023 PMC3285412

[b33] Pei Y. . An animal model of MYC-driven medulloblastoma. Cancer Cell 21, 155–167 (2012). 22340590 10.1016/j.ccr.2011.12.021 PMC3285431

[b34] Swartling F. J. . Pleiotropic role for MYCN in medulloblastoma. Genes Dev. 24, 1059–1072 (2010). 20478998 10.1101/gad.1907510 PMC2867210

[b35] Cho S. W., Kim S., Kim J. M. & Kim J. S. Targeted genome engineering in human cells with the Cas9 RNA-guided endonuclease. Nat. Biotechnol. 31, 230–232 (2013). 23360966 10.1038/nbt.2507

[b36] Jinek M. . RNA-programmed genome editing in human cells. eLife 2, e00471 (2013). 23386978 10.7554/eLife.00471 PMC3557905

[b37] Fu Y. . High-frequency off-target mutagenesis induced by CRISPR-Cas nucleases in human cells. Nat. Biotechnol. 31, 822–826 (2013). 23792628 10.1038/nbt.2623 PMC3773023

[b38] Hsu P. D. . DNA targeting specificity of RNA-guided Cas9 nucleases. Nat. Biotechnol. 31, 827–832 (2013). 23873081 10.1038/nbt.2647 PMC3969858

[b39] Pattanayak V. . High-throughput profiling of off-target DNA cleavage reveals RNA-programmed Cas9 nuclease specificity. Nat. Biotechnol. 31, 839–843 (2013). 23934178 10.1038/nbt.2673 PMC3782611

[b40] Cho S. W. . Analysis of off-target effects of CRISPR/Cas-derived RNA-guided endonucleases and nickases. Genome Res. 24, 132–141 (2014). 24253446 10.1101/gr.162339.113 PMC3875854

[b41] Jiang H. & Wong W. H. SeqMap: mapping massive amount of oligonucleotides to the genome. Bioinformatics 24, 2395–2396 (2008). 18697769 10.1093/bioinformatics/btn429 PMC2562015

[b42] Muguruma K. . Ontogeny-recapitulating generation and tissue integration of ES cell-derived Purkinje cells. Nat. Neurosci. 13, 1171–1180 (2010). 20835252 10.1038/nn.2638

[b43] Sato Y. . Stable integration and conditional expression of electroporated transgenes in chicken embryos. Dev. Biol. 305, 616–624 (2007). 17362912 10.1016/j.ydbio.2007.01.043

[b44] Guschin D. Y. . A rapid and general assay for monitoring endogenous gene modification. Methods Mol. Biol. 649, 247–256 (2010). 20680839 10.1007/978-1-60761-753-2_15

[b45] Zhang J., Kobert K., Flouri T. & Stamatakis A. PEAR: a fast and accurate Illumina Paired-End reAd mergeR. Bioinformatics 30, 614–620 (2014). 24142950 10.1093/bioinformatics/btt593 PMC3933873

[b46] Keane T. M. . Mouse genomic variation and its effect on phenotypes and gene regulation. Nature 477, 289–294 (2011). 21921910 10.1038/nature10413 PMC3276836

[b47] Robinson G. . Novel mutations target distinct subgroups of medulloblastoma. Nature 488, 43–48 (2012). 22722829 10.1038/nature11213 PMC3412905

[b48] Saeed A. I. . TM4: a free, open-source system for microarray data management and analysis. Biotechniques 34, 374–378 (2003). 12613259 10.2144/03342mt01

