# Supplementary material for: Somatic CRISPR/Cas9-mediated tumour suppressor disruption enables versatile brain tumour modelling
Source: Nat Commun. 2015 Jun 11;6:7391. doi: 10.1038/ncomms8391 (PMC4467376; doi:10.1038/ncomms8391)
Supplement: Supplementary Figures and Table — Supplementary Figures 1-10 and Supplementary Table 1 [file ncomms8391-s1.pdf]

Supplementary Figure 1
SURVEYOR assay

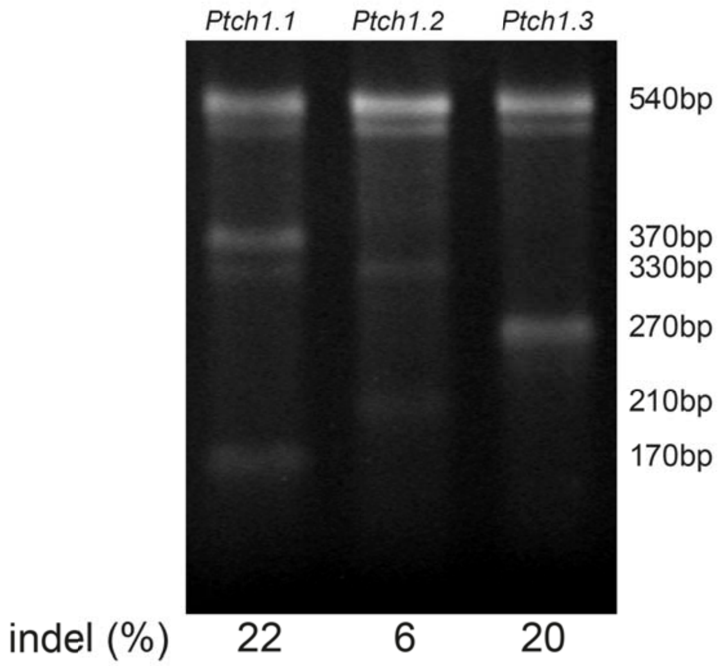

Supplementary Figure 1: Validation of gRNAs directed against the 2nd exon of the *Ptch1* gene using the SURVEYOR assay. Three different gRNAs (*Ptch1.1-Ptch1.3*) were tested for their efficiency of inducing indels. The upper bands indicate the unmodified PCR product (540 bp), the lower bands indicate SURVEYOR nuclease cleavage products (370-170 bp). Indel ratios were calculated as described in the methods section.

Supplementary Figure 2
P0 stereotactic cerebellar injections

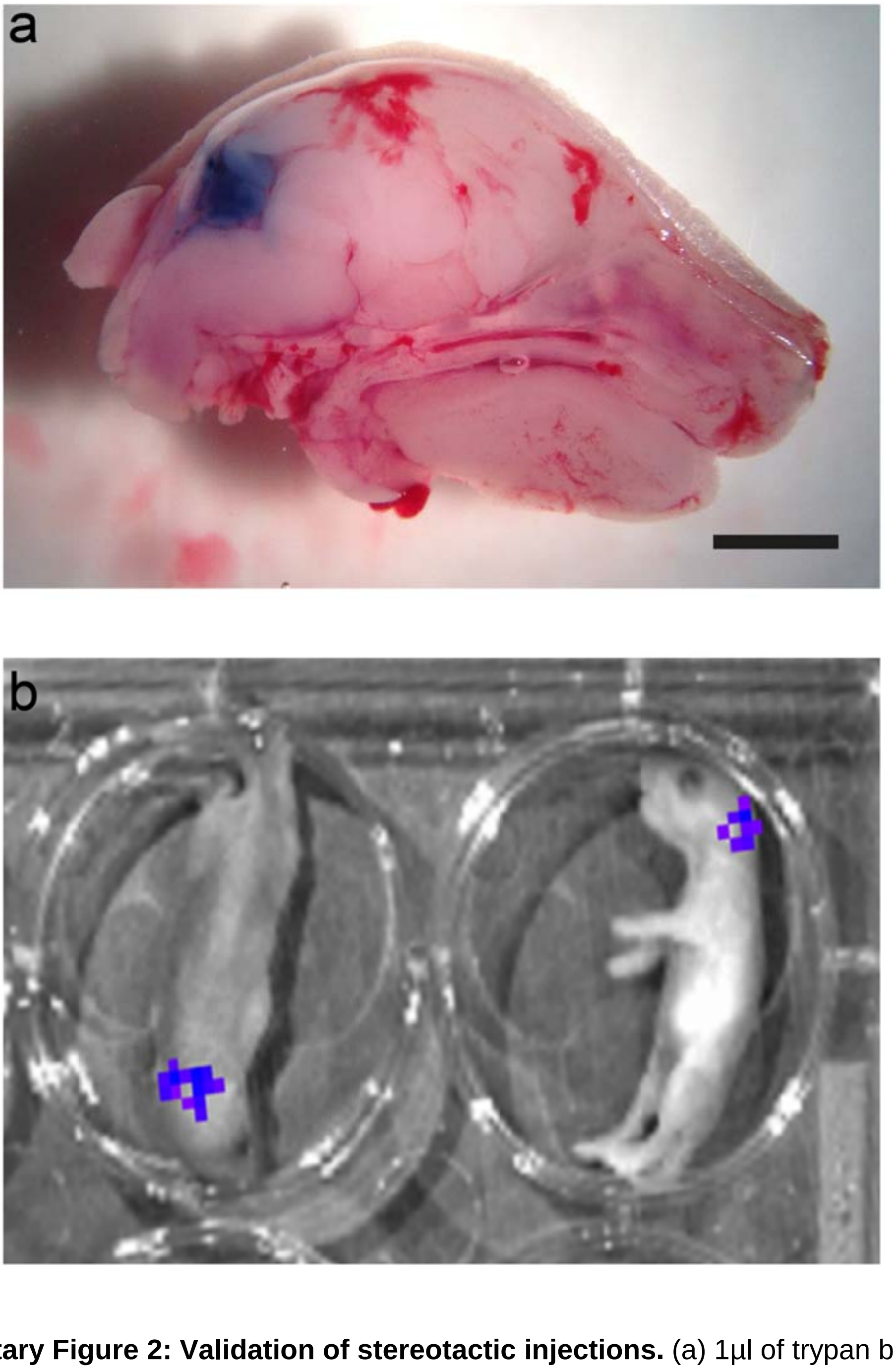

Supplementary Figure 2: Validation of stereotactic injections. (a) 1µl of trypan blue was injected to control for valid stereotactic coordinates. Sagittal section through the neonatal head showing successful injection into the cerebellum. Scale bar: 2mm. (b) Two days after PEI-mediated co-transfection of a plasmid encoding luciferase, transgene expression was assessed using intravital bioluminescence imaging.

Supplementary Figure 3

Sequencing after P0 PEI-mediated *gPtch1.1*/Cas9 transfection

a

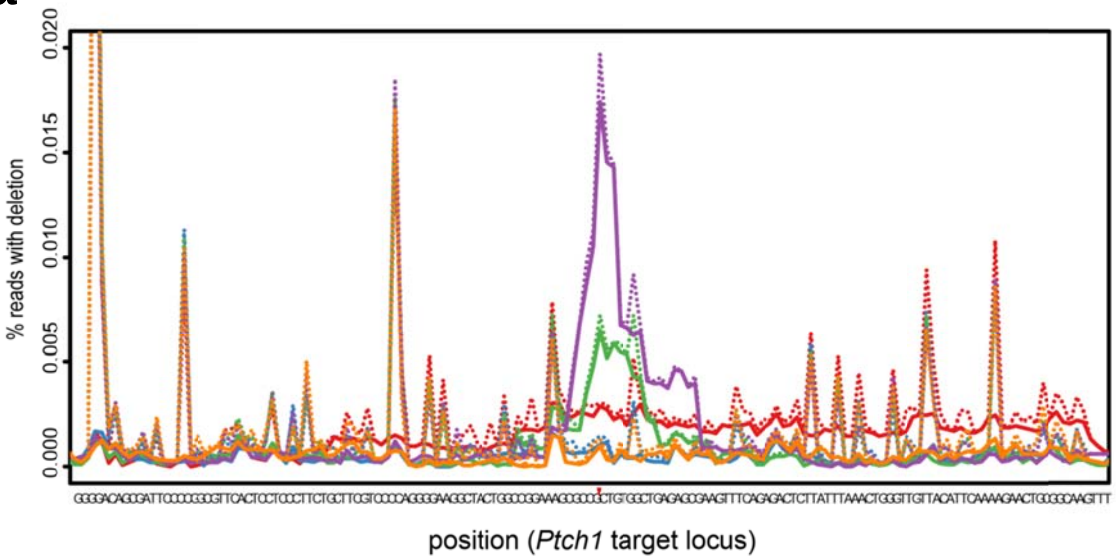

b

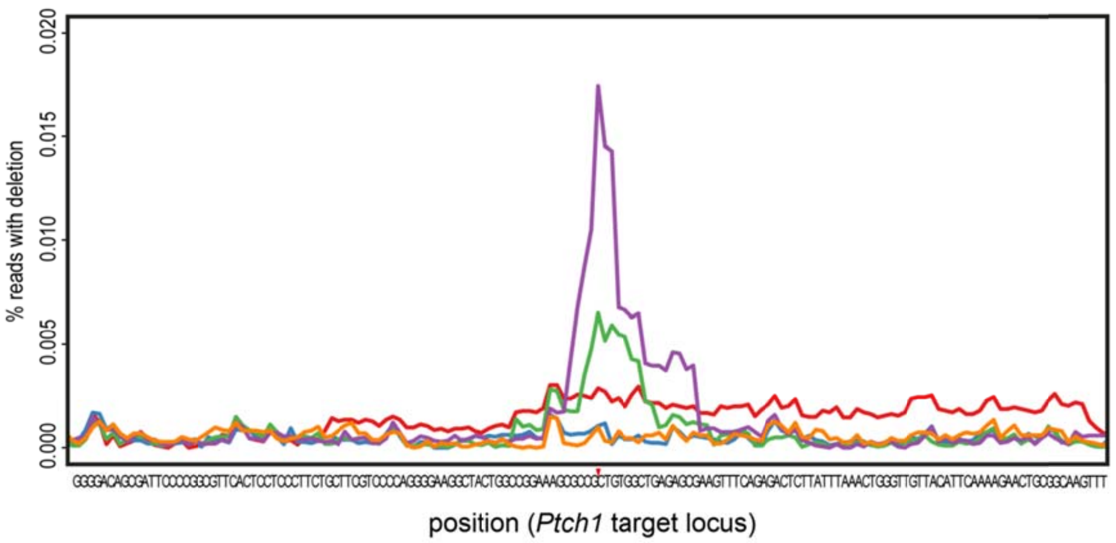

Supplementary Figure 3: Deep sequencing of the Ptch1 target locus from cerebella of 5 mice seven days after in vivo transfection of pX330 gPtch1.1/Cas9 plasmids. Percentages of reads with the specific base pair deletion indicated on the Y-axis were plotted against the sequence of the target locus. The raw data potentially include sequencing artifacts that usually affect single base pairs (dashed line, a). Therefore, single base pair deletions were omitted (b).

Supplementary Figure 4
Small proliferative neoplastic lesions found in
WT and *Trp53*$^{+/-}$ mice

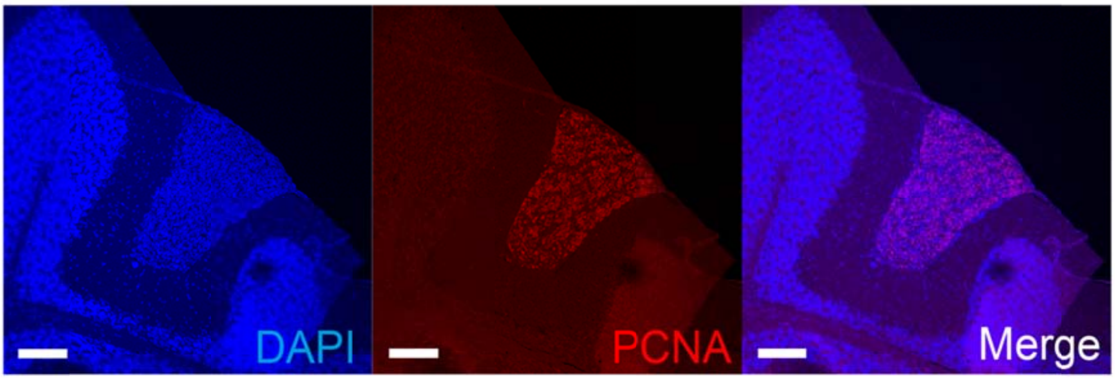

Supplementary Figure 4: Proliferative neoplastic lesions. Immunofluorescence analysis of small neoplastic lesions identified in WT and Trp53+/- animals after pX330 gPtch1/Cas9 injection. PCNA-positivity (red) demonstrates the proliferative state of these cells. Scale bars: 100µm.

Supplementary Figure 5
Tumor latencies

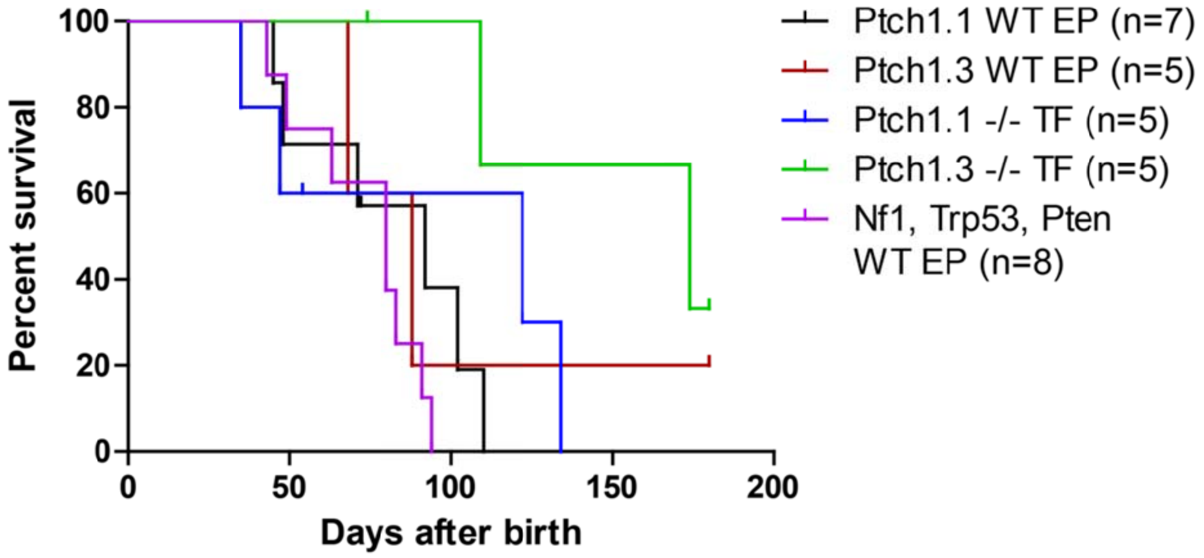

Supplementary Figure 5: Mouse survival after in utero electroporation or PEI transfection of indicated constructs. Kaplan-Meier plot of mice that developed neurological symptoms at the indicated time point, which were subsequently sacrificed and found to harbor an MB. The figure legend indicates the delivered gRNA, the genotype of transfected mice (wild type (WT) or Trp53-null (-/-)) and the approach used (EP = in utero electroporation; TF = PEI transfection). Mice sacrificed after control intervals are censored in this plot (vertical dashes).

Supplementary Figure 6
Cells targeted by cerebellar *in utero* EP

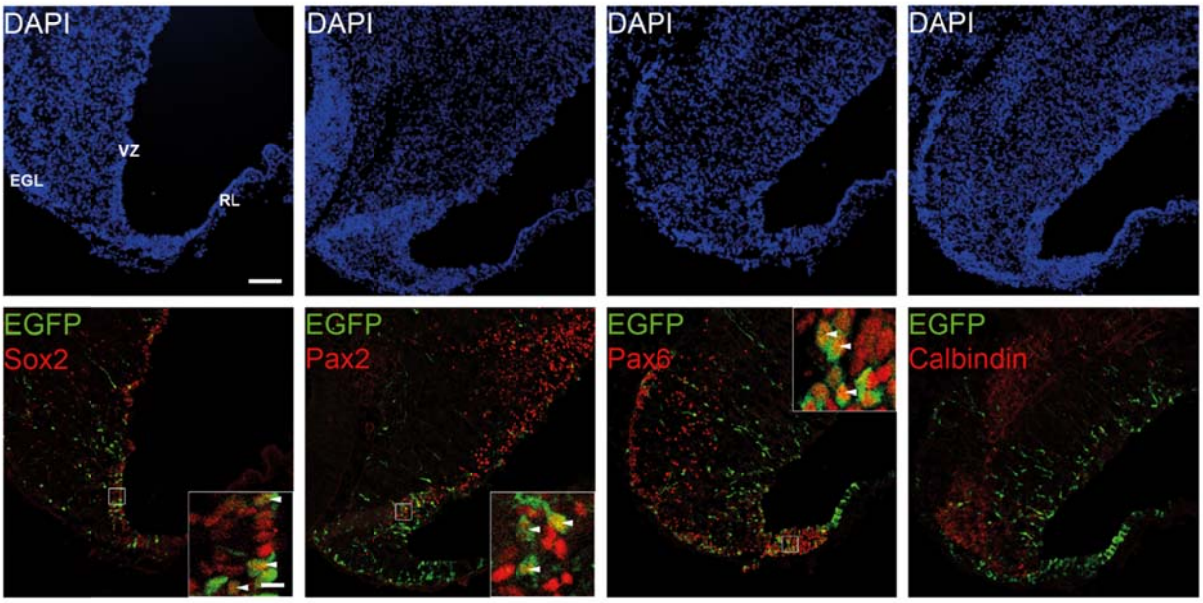

Supplementary Figure 6: Identification of cells targeted by in utero electroporation of GFP. Immunofluorescence analysis of murine cerebella at embryonic stage E15.5 two days after electroporation of a GFP expression vector showing that Pax2+, Pax6+ as well as Sox2+ cells can be targeted via in utero electroporation. Arrow heads indicate cells co-expressing GFP and the indicated marker. VZ = ventricular zone; RL = rhombic lip; EGL = external granule layer. Scale bars: 100µm (upper panel); 10µm (lower panel magnified insets).

Supplementary Figure 7
Validation of *in utero* electroporation by *in vivo* luciferase imaging

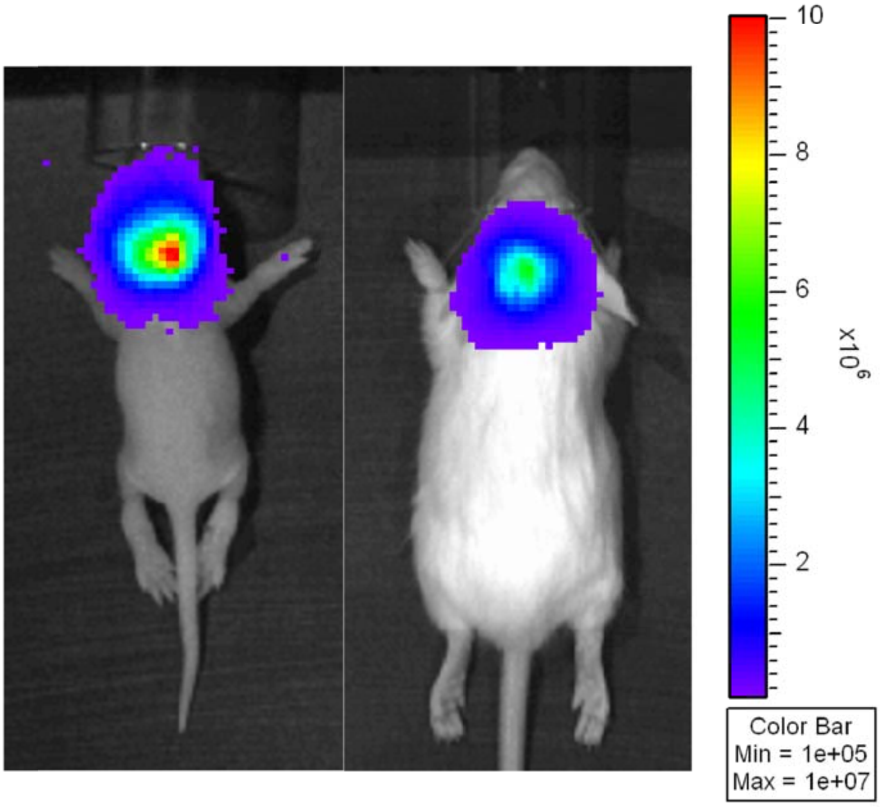

Supplementary Figure 7: Luciferase imaging after in utero electroporation. Cerebellar cells were co-electroporated with the pT2K-IRES-luciferase and pCAGGS-T2TP plasmids allowing for subsequent validation of successful electroporation and transgene expression. Animals were subjected to luciferase imaging at P2 (left mouse) and P20 (right mouse).

Supplementary Figure 8
Enlarged cerebellum after *gPtch1.1*/Cas9 *in utero* EP

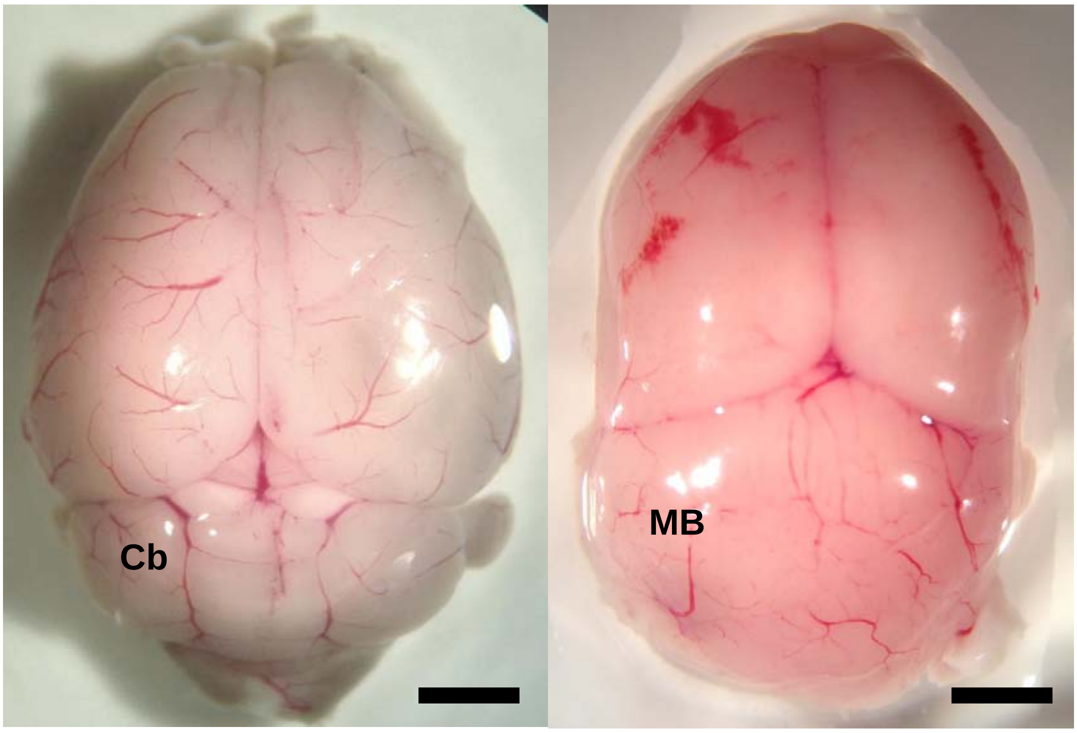

Supplementary Figure 8: Enlarged cerebellum after in utero electroporation of gPtch1/Cas9. Brains were dissected from either a WT mouse (left panel) or an animal electroporated with pX330 gPtch1/Cas9 that became symptomatic 7 weeks after birth (right panel). Cb = Cerebellum; MB = Medulloblastoma. Scale bars: 2.5mm.

Supplementary Figure 9
Ptch1 locus is disrupted in tumors induced with *gPtch1.1*/Cas9

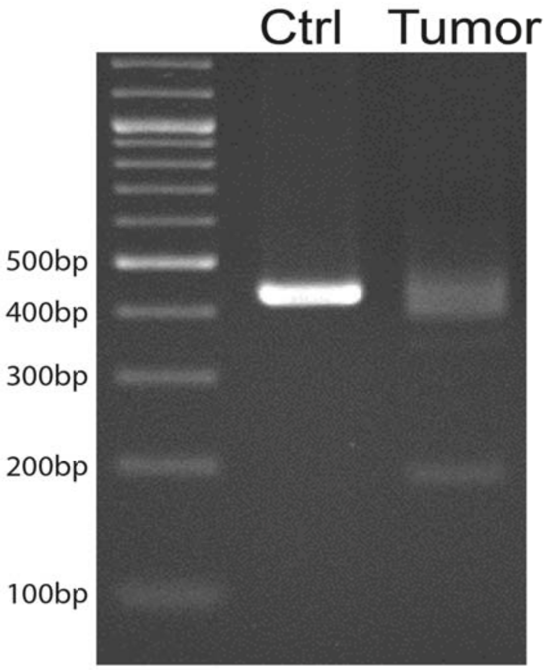

Supplementary Figure 9: The Ptch1 locus is disrupted in tumors induced with gPtch1/Cas9. The targeted locus was amplified from genomic DNA of normal brain control (Ctrl) or tumor tissue (Tumor) and analyzed on an agarose gel.

Supplementary Figure 10
H&E histology and immunofluorescence (IF) staining of small neoplastic lesions

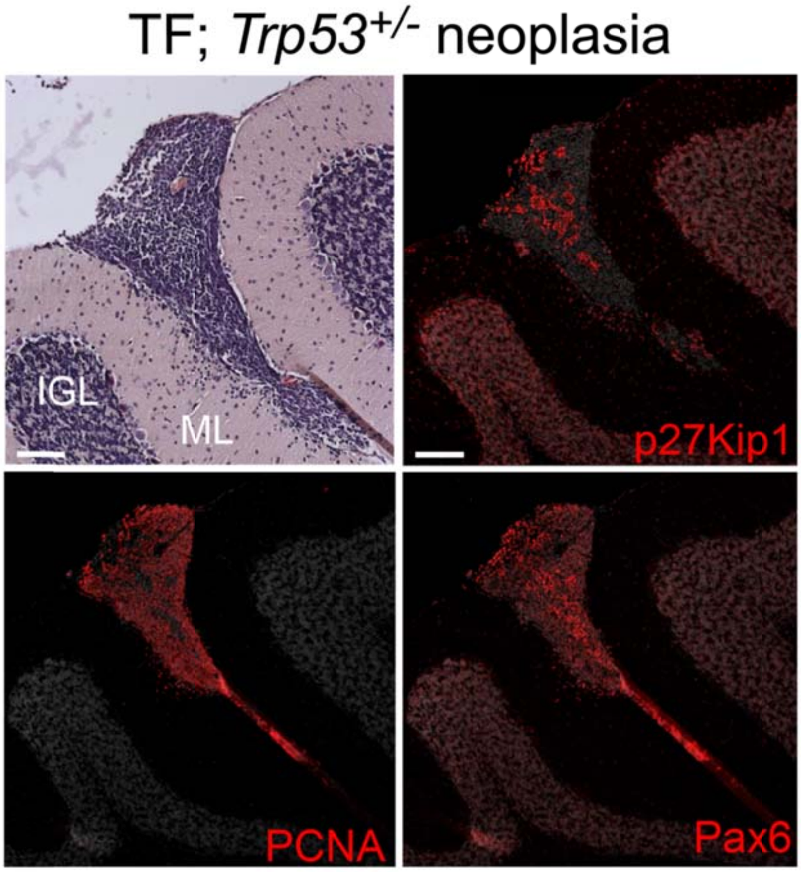

Supplementary Figure 10: H&E histology and immunofluorescence (IF) staining of a small neoplastic lesion induced in a Trp53+/- animal. The hypercellular lesion shows marked proliferation (PCNA) and expression of the granule cell marker Pax6. A fraction of cells have become postmitotic and stain positive for p27Kip1. (IGL, internal granule cell layer; ML, molecular layer). Scale bars: 100µm.

Supplementary Table 1
Mouse IDs

		Tumor	Method	*Trp53* genotype	Mouse_ID
Figure1	a	-	-	-	-
	b	MB	TF	WT	MZ7
	b	MB	TF	-/+	259
	c	MB	TF	-/-	261
	d	MB	EP	WT	MZ38
	e	MB	TF	WT	MZ7
	f	MB	TF	-/-	261
	g	MB	EP	WT	MZ38
Figure2	a,b	MB	TF	-/-	261, 248, 256
	a,b	MB	EP	WT	DK002, DK003, DK004
	c,d,e	MB	TF	-/-	261
	c,d,e	MB	EP	WT	DK002, DK003
	f	MB	TF	-/-	261
	f	MB	EP	WT	DK002
Figure3	a	GBM	EP	WT	MZ3_2
	b	GBM	EP	WT	MZ3_1
	c	GBM	EP	WT	MZ3_1
Supplementary Figure 4		MB	TF	WT	MZ7
Supplementary Figure 8		MB	EP	WT	MZ38
Supplementary Figure 9		MB	TF	WT	MZ7
Supplementary Figure 10		MB	TF	-/+	259
